# Associations of the Korean patient placement criteria matching among individuals with alcohol-related problems with treatment completion and abstinence: an observational study

**DOI:** 10.1186/s13722-024-00521-2

**Published:** 2024-12-26

**Authors:** Jiyoung Hong, Seon-Hi Shin, Ji Eun Kim, Sang Kyu Lee, Hong Seok Oh, Euihyeon Na, Hyun Ji Cho, Sungwon Roh

**Affiliations:** 1 Department of Psychiatry, Hanyang University Hospital, 222 Wangsimni-ro, Seongdong-gu, Seoul, 04763 Korea; 2 Biostatistics Laboratory, Medical Research Collaborating Center, Hanyang University, Seoul, Korea; 3 Department of Psychiatry, Hanyang University College of Medicine, Seoul, Korea; 4 Department of Psychiatry, Hallym University, Chuncheon Sacred Heart Hospital, Chuncheon, Korea; 5 Department of Psychiatry, Konyang University College of Medicine, Konyang University Hospital, Daejeon, Korea; 6 Department of Neuropsychiatry, Presbyterian Medical Center, Jeonju, Korea; 7 Department of Counseling Psychology, Sahmyook University, Seoul, Korea; 8 Department of Radiology, NYU Grossman School of Medicine, New York, USA

## Abstract

**Background:**

The American Society of Addiction Medicine Patient Placement Criteria (ASAM PPC) are guidelines for matching addiction patients to an optimal level of care (LOC). South Korea lacked a systematic approach to assigning alcohol use disorder patients to suitable treatment. To address this, Park et al. translated the ASAM PPC into Korean, creating the Korean Patient Placement Criteria (KPPC). We aim to assess the efficacy of the KPPC by evaluating whether receiving KPPC-matched treatment would result in longer periods of alcohol abstinence and higher number of treatment program completion.

**Methods:**

This is an observational, multi-site study of 225 individuals with hazardous alcohol use or alcohol dependence, defined by Alcohol Use Disorder Identification Test score of 10 or more for men, and 6 or more for women. We evaluated patients using KPPC at baseline and one-month follow-up visits and recommended a LOC at every visit. Patients freely chose to receive KPPC-matched treatment or not. We examined the duration of alcohol abstinence and number of one-month treatment program completion within a three-month period.

**Results:**

Of the 225 participants, 47 never pursued their matched level of care treatment, 54 pursued it once, and 124 pursued it twice. Individuals who received KPPC-matched treatment once had significantly higher odds of achieving alcohol abstinence (OR = 2.23), with greater odds when they received KPPC-matched treatments twice (OR = 2.88). The association was also significant for treatment completion, with greater odds of completing treatment program for one KPPC-matched treatment (OR = 3.28) and two KPPC-matched treatments (OR = 3.19).

**Conclusions:**

Individuals who followed the KPPC matched level of care had longer periods of alcohol abstinence and better treatment completion. Our results should encourage community addiction management centers and hospitals to adopt KPPC for classifying treatment settings for alcohol use disorder patients. Further research is warranted to maximize the potential benefits of KPPC.

**Supplementary Information:**

The online version contains supplementary material available at 10.1186/s13722-024-00521-2.

## Introduction

Harmful use of alcohol results in three million global deaths annually, representing 5.3% of all deaths [20]. Between 2017 and 2021, approximately 4900 alcohol-associated deaths occurred in South Korea annually, representing 9.5% of all deaths [22]. In 2021, the lifetime prevalence of alcohol use disorder (AUD) in South Koreans was 11.6%, highest among all mental disorders in the country [12]. However, only 3.2% of South Koreans diagnosed with AUD sought treatment, indicating a need to raise public awareness about AUD [12].

Interventions for high-risk groups in primary care settings can be effective in preventing AUD by reducing long-term treatment costs and alcohol consumption [9]. However, less than 20% of total cases were managed in optimal treatment settings. Most were managed simply by prescribing medications such as acamprosate and naltrexone rather than integrating alcohol rehabilitation in South Korea [11, 21]. There is a need for personalized, systematic treatment to improve AUD treatment.

The substance use disorder (SUD) treatment field has created intake tools that match patients to the most appropriate treatment setting. One of the best-known tools is the American Society of Addiction Medicine-Patient Placement Criteria (ASAM PPC) [16]. The ASAM PPC matches patients to suitable level of care (LOC) by evaluating addiction severity in six domains including mental, emotional, social, and medical conditions [6]. Previous studies have shown significantly lower alcohol consumption, lower treatment dropout rates, and improved outcomes in patients assigned to recommended LOC compared to those who were not [13, 14, 24, 25]. However, ASAM PPC are not yet widely accepted among community programs and hospitals in South Korea.

**Fig. 1:**
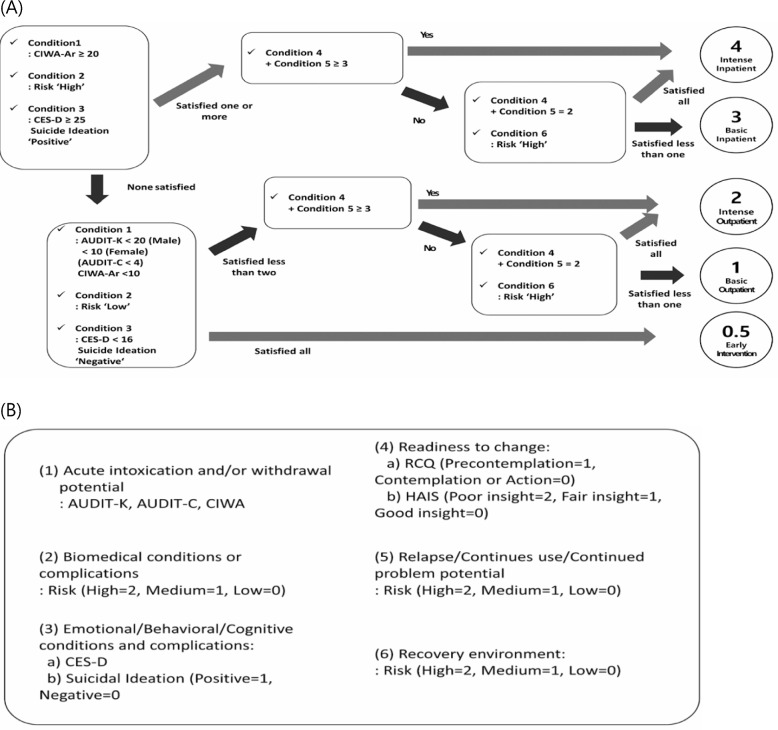
Final version of Korean Patient Placement Criteria (KPPC). Algorithm for assigning people to the levels of care (**A**) and 6 dimensions(conditions) of KPPC (**B**). Patients placement start by checking the condition scores in left upper box and work down the algorithm. Condition 4  + Condition 5 in (**A**) refers to addition of RCQ, HAIS and relapse risk score. CIWA-Ar: Clinical Institute Withdrawal Assessment for Alcohol, CES-D: Center for Epidemiologic Studies Depression Scale, AUDIT-K: Alcohol Use Disorder Identification Test-Korea, AUDIT-C: Alcohol Use Disorder Identification Test-Consumption Questiionnaire [18]

Until recently, there was no staging system in South Korea to assign patients with alcohol problems to suitable treatment [8]. To address this lack, the Korean PPC (KPPC) was adapted from the ASAM PPC by translating it into Korean (Fig. 1, [18]. Level 3 was further adapted from residential treatment to basic inpatient hospital treatment due to the scarcity of alcohol recovery centers and residential facilities in South Korea, while hospitals are sufficiently available. The KPPC has not been validated against the English version of the ASAM Criteria Software, known as CONTINUUM, which is the version officially endorsed by ASAM and which has undergone prior research validation [25]. We applied the KPPC to a sample of 225 individuals with hazardous alcohol use or alcohol dependence and compared alcohol abstinence and number of treatment completions between individuals who received adequate treatment (LOC as determined by KPPC or higher) and those who were undertreated (lower LOC than recommended).

## Methods

### Study population

A total of 23 hospitals participated in this study, including eight specialized hospitals for alcohol treatment (Dasarang Central Hospital, Aju Pyeonhan Hospital, Jin Hospital, W Jin Hospital, Dasarang Hospital, Hansarang Hospital, Yesarang Hospital and Onsarang Hospital), six general hospitals (Hanyang University Hospital, Konyang University Hospital, Hallym University Chuncheon Sacred Heart Hospital, Yangsan Pusan National University Hospital, Jesus Hospital, and Daejeon Eulji University Hospital), and nine psychiatric hospitals (Bugok National Hospital, Jeonbuk Maumsarang Hospital, Incheon Chamsarang Hospital, Yonkang Hospital, With Hospital, Hyeongju Hospital, Cheonju St. John Hospital, Haenam Hyemin Hospital, and Daedong Hospital). All participating hospitals offered the 4 levels of care except for level 0.5. Patients with level 0.5 were referred to community center for treatments. All patients began treatment at one of the 23 hospitals and each of the 23 hospitals treated at least one patient. Individuals with alcohol problems who visited these hospitals from March 20th, 2020, to December 31, 2021, and were newly entering treatment for an AUD were invited to participate in this study. People who agreed to participate were screened using the self-reported Alcohol Use Disorder Identification Test-Korean (AUDIT-K) [10]. Males with score of 10 or more and females with score of 6 or more were eligible for enrollment. Participants had to be ≥ 18 years of age with no confirmed history of brain damage, organic mental disorder, or suspected intellectual disability. All subjects were informed of the study purpose, contents, and potential risks and provided written consent. The study was approved by the institutional review boards (IRBs) of all participating institutes (IRB NO. HYUH 2020-01-032, IRB NO. 2019-02-010, IRB NO. 2020-03-010, IRB NO. 206-82-07306, IRB NO. 05–2021-007).

### Study procedure

Enrolled participants underwent evaluations through self-report questionnaires and computer-facilitated intake assessments based on the KPPC at baseline and 1-month follow up. The questionnaires included questions about demographic characteristics (sex, education, occupation, marital status, socioeconomic status (SES)), AUDIT-K, AUDIT-Consumption (AUDIT-C), the Center for Epidemiologic Studies-Depression Scale (CES-D), Hanil Alcohol Insight Scale (HAIS), Readiness to Change Questionnaire (RCQ), and the Clinical Institute Withdrawal Assessment of Alcohol Scale, Revised (CIWA-Ar). Physical health condition, risk of relapse, and environmental risks were accessed through structured clinical interviews by case managers.

**Fig. 2:**
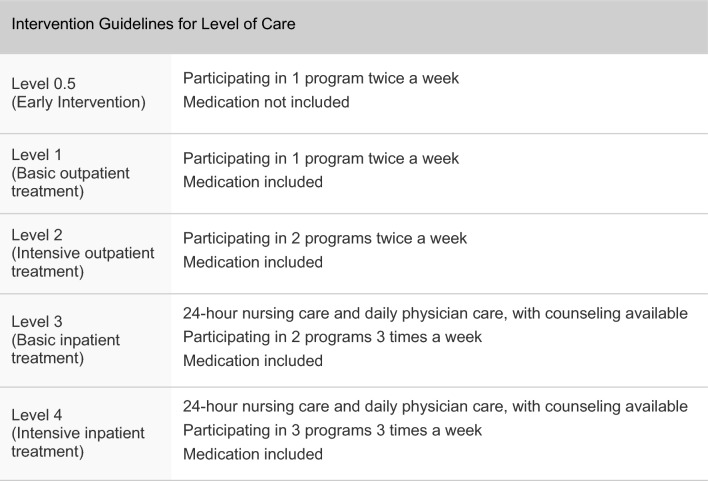
Interventions guideline for level of care. Programs for level 0.5 include sobriety program and community center-based case management. Medication include acamprosate, naltrexone, antidepressants and anxiolytics. Programs for level 1,2,3,4 include motivational enhancement therapy, cognitive behavioral therapy, 12-step programs, relapse prevention education, disease education, family therapy, hospital-based case management, community center-based case management, therapeutic communities, and Alcoholics Anonymous

Each participant’s LOC was determined at every assessment, ranging from level 0.5 to 4 (0.5, 1, 2, 3, 4) [18]. These respective levels indicate early intervention (0.5), basic outpatient (1), intensive outpatient (2), basic inpatient (3), and intensive inpatient (4) treatment. Based on the LOC, participants were guided to receive interventions that could include medications (including naltrexone, acamprosate, antidepressants and anxiolytics), motivational enhancement therapy, cognitive behavioral therapy, 12-step programs, relapse prevention education, disease education, family therapy, hospital-based case management, community center-based case management, therapeutic communities, or Alcoholics Anonymous (Fig. 2). Some patients also utilized the Addiction Support Center, a resource within the South Korean substance use care system which offers assistance with diagnosis, access to economic resources, Alcoholics Anonymous meetings, and rehabilitation services. Case managers provided one to four counseling sessions per month to assist and engage subjects in treatment.

Participants freely chose to receive the recommended LOC or not at every assessment. We categorized individuals who received treatment at the recommended LOC or higher as KPPC-matched and those who received lower level treatment as KPPC-mismatched. We compared patients regarding duration of alcohol abstinence and number of treatment completions according to the number of KPPC-matched treatments they received: 0, 1, or 2.

### Measures

#### Primary outcomes

The outcomes were assessed at 1-month and 3-months follow up. Duration of alcohol abstinence was determined by self-reported AUDIT-C score of 0, indicating one month of no alcohol. We assessed the AUDIT-C score at one- and three-month follow-up and summed up the number of months with no alcohol for each participant—0, 1, or 2. Individuals who missed the second AUDIT-C assessment at the 3-month follow up were considered to be not alcohol abstinent for the month. Treatment program completion was defined as successful conclusion of a 30-day treatment program. The number of months with successful treatment completion was recorded—0, 1, 2, or 3 out of three-months. We also conducted separate analyses to determine if the effect of adhering to KPPC recommendations varied based on whether the first referral was for inpatient or outpatient care.

#### Independent variables

We used the KPPC to decide patients’ LOC which includes multiple scales and questionnaires in six dimensions: (1) Intoxication and Withdrawal (2) Biomedical Conditions and Complications, (3) Emotional or Behavioral Conditions, (4) Treatment Readiness, (5) Potential for Relapse, and (6) Environmental Conditions (Fig. 1, [18]). The scales included were AUDIT-K, AUDIT-C, CES-D, RCQ, HAIS, and CIWA-Ar. Besides the scales, the assessment included participants’ subjective report on self-health, diagnosis and treatment history, presence of suicidal ideation, use of community addiction management center, drug compliance, subjective report of craving, and environmental factors such as family, occupation, friends, religion, and financial status. KPPC was evaluated twice—at baseline and 1-month follow-up. We compared the participants by the number of times they received KPPC-matched treatments—0, 1 and 2.

#### Control variables

The control variables included biological sex, employment status, and previous hospitalization for AUD. These factors were controlled because male gender is associated with a higher risk of lifetime AUD [7], and being employed is linked to better abstinence outcomes in individuals with AUD [5]. Hospitalizations are known to promote behavior change by initiating medication for AUD [15], which might have positive effect on the outcomes.

### Statistical analysis

Multiple contingency tables were formed using the number of KPPC-matched treatments and each of the other variables. Subsequently, frequency analysis followed by the Chi-square or Mantel–Haenszel Chi-square test was performed to examine the baseline characteristics of study participants and to gauge the effects of the KPPC-matched treatments on primary outcomes. These variables were also provided with Cramer's V as the effect size. For the primary outcome, a post-hoc power analysis was conducted using GPower software [3]. The effect size used in G*Power version 3.1 [4] was w, calculated as w = Cramer’s V × √(r-1), where r represents the number of categories in the smaller variable of the contingency table [3].

We conducted ordinal logistic regression analyses with an unadjusted model and an adjusted model that added potential confounders to the unadjusted model [1]. The predictor variable of primary interest was the number of KPPC-matched treatments, and the confounders were biological sex, employment status, and prior hospitalization for AUD.

For each outcome variable which was measured on an ordinal scale, the satisfaction of the proportional odds assumption was checked before attempting to interpret the results of the two ordinal logistic regression models. Additionally, to identify the better model, the likelihood ratio test based on -2LLs from the two models was performed while the model fit statistics, AIC and SC were also compared between the models [19].

For each model, odds ratios (ORs) and 95% confidence intervals (CIs) were examined to judge substantive significance and *p* values were reviewed for statistical significance. Quasi-complete separation [23] was also checked to ensure the integrity of the analysis results.

Meanwhile, the same ordinal logistic regression models were separately fitted for subgroups based on inpatient and outpatient care to differentiate between referrals to inpatient and outpatient settings.

## Results

### Subject characteristics

**Fig. 3:**
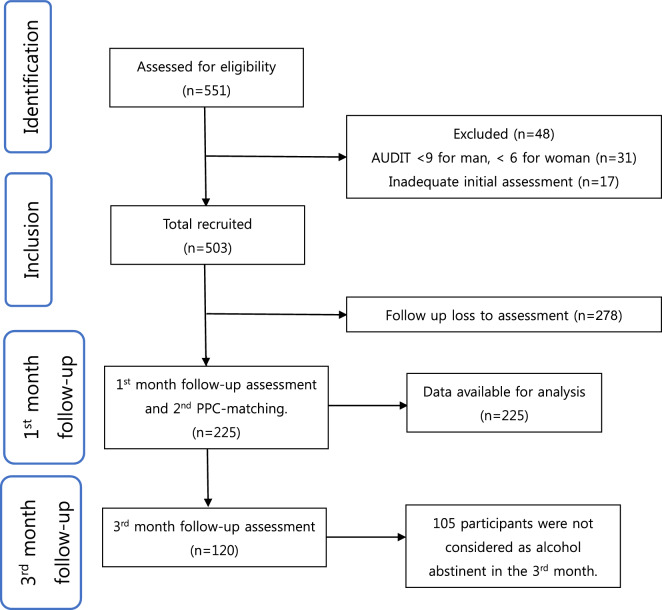
STROBE flowchart of participants in this study

A total of 551 individuals were assessed and 503 were eligible. Of these, 281 were lost to follow-up meaning they did not stay in treatment until the second KPPC assessment, leaving 225 participants for analysis (Fig. 3). Among the 225 participants who underwent 2 KPPC-matchings, 105 missed the AUDIT-C assessment at the 3-month follow up, and were considered as not alcohol abstinent for the month. As shown in Table 1, the study included 161 (71.6%) males and 64 (28.4%) females. Men were more likely to never choose a matched level of care than women. Employment was reported by only 41 (18.2%) participants. Among the subjects, 80 (35.6%) had a history of previous admission due to AUD. 81.3% of individuals had a high school education or higher, and 55.5% belonged to the middle and upper socioeconomic classes. Only 18% of patients utilized community addiction management centers. Marital status was evenly distributed. At baseline assessment, 89 (39.6%) participants were recommended to outpatient care (LOCs 0.5, 1, and 2), while 136 (60.4%) were recommended inpatient care (LOCs 3 and 4). Participants categorized to outpatients were more likely to choose a matched level of care. The effect sizes for the statistically significant variables ranged from small to medium.

**Table 1:** Characteristics of study participants

Variables	All (*n* = 225)	No. of KPPC-matched treatments			${\chi }^{2}$	*Cramer’s V*
		0 (*n* = 47)	1 (*n* = 54)	2 (*n* = 124)		
Sex					6.77^*^	0.17
Male	161 (71.6%)	40 (85.1%)	40 (74.1%)	81 (65.3%)		
Female	64 (28.4%)	7 (14.9%)	14 (25.9%)	43 (34.7%)		
Marital status					2.31	0.07
Single	63 (28.6%)	13 (28.9%)	15 (28.9%)	35 (28.5%)		
Married	87 (39.5%)	15 (33.3%)	24 (46.2%)	48 (39.0%)		
Separated/divorced/bereaved	70 (31.8%)	17 (37.8%)	13 (25.0%)	40 (32.5%)		
Missing	5					
Employment status					6.74^*^	0.17
Employed	41 (18.2%)	14 (29.8%)	11 (20.4%)	16 (12.9%)		
Unemployed	184 (81.8%)	33 (70.2%)	43 (79.6%)	108 (87.1%)		
Education					0.04	0.13
Less than middle school	16 (7.3%)	5 (11.1%)	0 (0%)	11 (8.9%)		
Middle school	21 (9.5%)	4 (8.9%)	4 (7.7%)	13 (10.6%)		
High school	105 (47.7%)	23 (51.1%)	28 (53.9%)	54 (43.9%)		
More than high school	78 (35.5%)	13 (28.9%)	20 (38.5%)	45 (36.6%)		
Missing	5					
Previous hospitalization due to AUD					4.13	0.14
Yes	80 (35.6%)	12 (25.5%)	17 (31.5%)	51 (41.1%)		
No	145 (64.4%)	35 (74.5%)	37 (68.5%)	73 (58.9%)		
SES^†^					0.40	0.10
Lower class	97 (43.7%)	22 (47.8%)	19 (35.9%)	56 (45.5%)		
Middle class	95 (42.8%)	20 (43.5%)	28 (52.8%)	47 (38.2%)		
Upper class	30 (13.5%)	4 (8.70%)	6 (11.3%)	20 (16.3%)		
Missing	3					
Using the Addiction Support Center^+^					3.23	0.12
Yes	40 (17.8%)	7 (14.9%)	14 (25.9%)	19 (15.3%)		
No	185 (82.2%)	40 (85.1%)	40 (74.1%)	105 (84.7%)		
Baseline recommended LOC					19.68^***^	0.30
Outpatient (levels 0.5, 1, 2)	89 (39.6%)	13 (27.7%)	11 (20.4%)	65 (52.4%)		
Inpatient (levels 3, 4)	136 (60.4%)	34 (72.3%)	43 (79.6%)	59 (47.6%)		

The post-hoc power analysis using G*Power software assessed the robustness of the significance of the KPPC-matched treatment effect on alcohol abstinence and treatment completion. The effect size used in G*Power was *w* = Cramer’s V × $\sqrt{r-1}$, where *r* represents the number of categories in the smaller variable of the contingency table [3]

^+^The Addiction Support Center is a community center that supports people with substance use disorders. Patients visit the center for assistance with diagnosis, economic resources, Alcoholics Anonymous meetings, and rehabilitation

^†^The Mantel–Haenszel Chi-square test was used because the row and column variables are on an ordinal scale

^*^p < 0.05 ^**^p < 0.01 ^***^p < 0.001

Meanwhile, as shown in Table 2, the associations between the number of KPPC-matched treatments and alcohol abstinence, as well as treatment completion, were statistically significant, with small effect sizes. The post-hoc power analysis was conducted to assess the robustness of the significance of the KPPC-matched treatment effect on alcohol abstinence and treatment completion. The resulting powers for alcohol abstinence and treatment completion were approximately 0.45 and 0.64, respectively.

**Table 2:** The results of significance tests and effect sizes

Variables	All (*n* = 225)	No. of KPPC-matched treatments			${\chi }^{2}$	*Cramer’s V*
		0 (*n* = 47)	1 (*n* = 54)	2 (*n* = 124)		
Duration of alcohol abstinence in months^†^					9.73^**^	0.16
0	98 (43.6%)	30 (36.8%)	24 (44.4%)	44 (35.5%)		
1	79 (35.1%)	12 (25.5%)	18 (33.3%)	49 (39.5%)		
2	48 (21.3%)	5 (10.6%)	12 (22.2%)	31 (25.0%)		
No. of one-month treatment completion^†^					13.27^***^	0.21
0	102 (45.3%)	32 (68.1%)	23 (42.6%)	47 (37.9%)		
1	18 (8.00%)	6 (12.8%)	3 (5.6%)	9 (7.3%)		
2	37 (16.4%)	4 (8.5%)	8 (14.8%)	25 (20.2%)		
3	68 (30.2%)	5 (10.6%)	20 (37.0%)	43 (34.7%)		

The post-hoc power analysis using G*Power software assessed the robustness of the significance of the KPPC-matched treatment effect on alcohol abstinence and treatment completion. The effect size used in G*Power was *w* = Cramer’s V × $\sqrt{r-1}$, where *r* represents the number of categories in the smaller variable of the contingency table [3]. The resulting powers for alcohol abstinence and treatment completion were approximately 0.45 and 0.64, respectively

^†^The Mantel–Haenszel Chi-square test was used because the row and column variables are on an ordinal scale

^*^p < 0.05 ^**^p < 0.01 ^***^p < 0.001

We conducted an additional analysis to compare the baseline LOC recommendation percentages between patients who were lost to follow-up and those who were successfully followed up. Female were more likely to follow up than male participants. Although the follow-up lost group showed a higher likelihood of being recommended to Level 4 care, this difference was not statistically significant (Online Appendix Table 1).

### Treatment outcomes

#### Alcohol abstinence

**Fig. 4:**
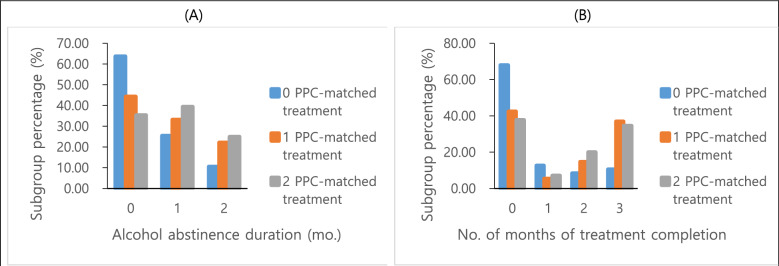
Subgroup percentages (%) of no. of PPC-matched treatment by alcohol abstinence duration (**A**) and no. of months of treatment completion (**B**). (*n*=225)

Individuals who received 1 KPPC-matched treatment showed higher odds of alcohol abstinence compared to the 0 KPPC-matched group (OR = 2.29[1.06–4.95]; *p* = 0.03) (Table 3). The odds ratios became higher (OR = 3.08[1.57–6.04]; *p* < *0.01*) for participants who received 2 KPPC-matched treatments (Table 3, Fig. 4A). When confounders were added to the model, the odds ratios remained significant, but their magnitude became slightly lower (OR = 2.23 [1.03–4.86] and 2.88 [1.44–5.76]; *p* = 0.04 and *p* < 0.01, respectively). Among the confounders "Previous hospitalization due to AUD" was statistically significant.

**Table 3:** Association of KPPC-matched treatment with the duration of alcohol abstinence with and without adjustment for confounders (*n* = 225)

Predictor variable and category vs reference category	Odds ratio [95% CI]	*p*	Fit statistics		
			AIC	SC	−2LL
No. of KPPC-matched treatments					
Unadjusted model^a^			473.15	486.82	465.15
Once vs none	2.29 [1.06–4.95]	0.03			
Twice vs none	3.08 [1.57–6.04]	< 0.01			
Adjusted model^b^					
Once vs none	2.23 [1.03–4.86]	0.04	471.71	495.62	457.71
Twice vs none	2.88 [1.44–5.76]	< 0.01			
Sex					
Male vs female	1.31 [0.74–2.30]	0.35			
Employment status					
Employed vs unemployed	0.64 [0.32–1.28]	0.21			
Previous hospitalization due to AUD					
Yes vs no	1.75 [1.04–2.97]	0.04			

Two ordinal logistic regression models satisfied the proportional odds assumption at the significance level of 0.05. Although the model-fit statistic AIC was slightly smaller in the adjusted model, the likelihood ratio test based on -2LLs from the two models was non-significant, and the SC was smaller in the unadjusted model

^a^The unadjusted model includes only the independent variable, *No. of KPPC-matched treatments*

^b^The adjusted model adds *Sex, Employment status, and Previous hospitalization due to alcohol use disorder*

Meanwhile, all four models for both subgroups referred to outpatient or inpatient care in the first match satisfied the proportional odds assumption and encountered no issues of quasi-complete separation. However, some estimates' odds ratios (ORs) and their standard errors were unusually large, possibly due to a small sample size. Therefore, caution should be exercised in interpreting the results. The 2 KPPC-matched group showed a significant OR of 2.88 (p < 0.01) for outpatients in the adjusted model (Online Appendix Tables 2, 3). The results for inpatients were not significant in both adjusted and unadjusted models.

#### Treatment completion

Individuals who received KPPC-matched treatment showed higher odds of treatment completion. The odds ratio for 1 KPPC-matched treatment compared to 0 KPPC-matched treatment was 3.54 (95% CI: 1.61–7.76; *p* < 0.01) Table 4). This trend was more prominent for the group that received 2 KPPC-matched treatments (OR = 3.76 [1.87–7.53]; *p* < 0.001). Figure 4B). When confounders were added to the model, the odds ratios remained significant (OR = 3.28 [1.48–7.26] and 3.19 [1.56–6.52]; *p* < 0.01 and *p* < 0.01, respectively). None of the confounders were statistically significant.

**Table 4:** Association of KPPC-matched treatment with the number of months of treatment completion with and without adjustment for confounders (*n* = 225)

Predictor variable and category vs reference category	Odds ratio [95% CI]	*p*	Fit statistics		
			AIC	SC	−2LL
No. of KPPC-matched treatments					
Unadjusted model^a^			541.98	559.06	531.98
Once vs none	3.54 [1.61–7.76]	< 0.01			
Twice vs none	3.76 [1.87–7.53]	< 0.001			
Adjusted model^b^					
Once vs none	3.28 [1.48–7.26]	< 0.01	541.62	568.95	525.62
Twice vs none	3.19 [1.56–6.52]	< 0.01			
Sex					
Male vs female	0.67 [0.38–1.17]	0.16			
Employment status					
Employed vs unemployed	0.65 [0.32–1.30]	0.22			
Previous hospitalization due to AUD					
Yes vs no	1.42 [0.84–2.40]	0.19			

Two ordinal logistic regression models satisfied the proportional odds assumption at the significance level of 0.05. None of the confounders were significant. Although the model-fit statistic AIC was slightly smaller in the adjusted model, the likelihood ratio test based on −2LLs from the two models was non-significant, and the SC was smaller in the unadjusted model

^a^The unadjusted model includes only the independent variable, *No. of KPPC-matched treatments*

^b^The adjusted model adds *Sex, Employment status, and Previous hospitalization due to alcohol use disorder*

Some of the four models for both subgroups referred to outpatient or inpatient care in the first match failed to satisfy the proportional odds assumption, although there was no issue of quasi-complete separation. Some estimates' odds ratios (ORs) and their standard errors were large, potentially due to a small sample size. The odds ratios for inpatients were significant in the adjusted model: 2.73 (p = 0.02) for those who received KPPC-matched treatment once compared to those who received no treatment. The significance was even greater for patients who received KPPC-matched treatment twice, with an odds ratio of 3.77 (p < 0.01) (Online Appendix Tables 4, 5). The results for outpatients were not significant in the adjusted model.

## Discussion

Individuals who received KPPC-matched treatment had higher likelihood of abstaining from alcohol (OR = 2.23). This association was more prominent with repeated treatment (OR = 2.88). Individuals who received KPPC-matched treatment also showed higher odds of treatment completion. People who received one KPPC-matched treatment had a higher odds ratio of completing treatment for one month (OR = 3.28) than people who received lower level treatment. This trend was similar for the group that received two KPPC-matched treatments (OR = 3.19). In subgroup analyses of inpatient versus outpatient referrals, more matched treatments showed higher odds of treatment completion. However, only the outpatient group with two KPPC-matched treatments showed a significant result for the duration of alcohol abstinence. We believe this is due to the significantly smaller sample size compared to the entire population, which resulted in very large standard errors.

Baseline characteristics of study participants as shown in Table 1 indicates that being employed was negatively associated with matching, likely due to concerns about missing work. Given that a significant proportion of patients (43.7%) are from lower socioeconomic backgrounds, extended treatment admissions of one month or longer could result in job loss and financial instability, highlighting the need for financial and vocational support in AUD treatment. Additionally, we expected previous hospitalization to be linked to a higher likelihood of accepting ASAM LOC recommendations, but this was not significant. Instead, previous hospitalizations were associated with longer durations of alcohol abstinence (OR = 1.75, p = 0.04). This suggests that Motivational Enhancement Therapy (MET) and medication during previous admission might help patients better understand their diagnosis and promote behavior change, leading to improved outcomes.

Our findings might imply that following the LOC determined by KPPC-based assessment can help subjects complete treatment and result in longer periods of alcohol abstinence. However, patients may resist the recommended LOC due to poor insights, financial burdens, inability to take extended sick leave, or fear of hospitalization, which is often portrayed negatively in the media. Lower LOC may provide insufficient treatment, leaving patients with inadequate education about their disease and treatment. Individuals with SUD may receive less motivation in these settings, despite research showing that at least three months of treatment is typically needed to reduce or stop substance use [17]. Unfortunately, 31% of patients discontinue SUD treatment early, with the majority dropping out within the first month [27].

To our knowledge, there are no previous studies examining the associations of KPPC with duration of alcohol abstinence and treatment completion. This study is notable for its specificity regarding AUD, which provide important insights into the effectiveness of KPPC-based assessment in alcohol abstinence.

This study has several limitations. First, due to the non-randomized design of the study, causal conclusions cannot be drawn, and outcome measures are likely influenced not only by the LOC treatment but also by patient motivation, which may confound the results. To address this issue, we controlled employment status, and prior hospitalization for AUD in our adjusted model, which turned out to be not significant. We could not randomly assign patients because our treatment included inpatient treatment and we could not force people to be hospitalized or not against their will. In this circumstance, we tried to minimize the possibility of external factors interfering with their choice and made sure subjects’ preferred treatment were always open to patients. Second, the post-hoc power was approximately 0.45 and 0.64, respectively, indicating that the study may not have had a large enough sample size to achieve high power. Future studies with a sufficiently large sample size are needed, and more valid conclusions can be drawn through replications. Third, alcohol abstinence was measured solely by self-reported AUDIT scores without objective tests such as blood alcohol concentration because it is difficult to measure alcohol concentrations of outpatients daily. While self-report data are subject to bias, AUDIT is a validated instrument for assessing alcohol use. Fourth, we aggregated KPPC-matched and over-matched patients due to insufficient sample size for repeated treatment categorization. Within our treatment setting, the predominant concern was undertreatment rather than overtreatment and thus, our study focused on treatment outcomes over considerations of cost-efficiency. Two prior studies reported that over-matching was associated with adverse clinical outcomes such as increased no-show rates and less reduction in Addiction Severity Index Composite Scores [2, 25, 26]. If over-matching had been specifically examined, even greater benefits of matching might have been found. Lastly, the study excluded patients with history of brain damage, organic mental disorder, or suspected intellectual disability and results may have been different with the inclusion of such patients.

Before the introduction of the KPPC, patients were diagnosed clinically, and treatment decisions were made by psychiatrists primarily focused on determining the need for hospitalization, with no detailed classification based on the severity of the patient's condition. Fortunately, KPPC has been adopted in some hospitals and Addiction Support Centers. By implementing the KPPC, patients can receive personalized treatment and better comprehend the need for treatment, which is especially crucial for patients with AUD due to their poor compliance. Rather than subjecting patients to generalized treatment programs or requiring hospitalization without empirical evidence, providing patients with KPPC assessments can significantly increase patient engagement [13, 14].

## Conclusions

Following the LOC based on KPPC was associated with increases in the treatment program completion and alcohol abstinence. This finding could motivate community addiction management centers and hospitals to incorporate KPPC for determining treatment approaches for individuals with alcohol addiction. Further studies with a randomized controlled design would help confirm the effectiveness of KPPC-based treatment and identify strategies to optimize its advantages.

## Supplementary Information

Supplementary Material 1.

Supplementary Material 2.

Supplementary Material 3.

## Data Availability

The datasets used and/or analysed during the current study are available from the corresponding author on reasonable request.
