# Supplementary material for: Associations of the Korean patient placement criteria matching among individuals with alcohol-related problems with treatment completion and abstinence: an observational study
Source: Addict Sci Clin Pract. 2024 Dec 26;19:98. doi: 10.1186/s13722-024-00521-2 (PMC11670465; doi:10.1186/s13722-024-00521-2)
Supplement: Supplementary file 1 — Supplementary Material 1. [file 13722_2024_521_MOESM1_ESM.docx]

Appendix Table 1. Characteristics of study participants including follow-up loss group

| Variables | All (*n* = 503) | Follow-up vs. Follow-up Loss group | | ${}^{2}$ |
| --- | --- | --- | --- | --- |
|  |  | Follow-up  (*n* = 225) | Lost to follow-up  (*n* = 278) |  |
| Sex |  |  |  | 6.65^**^ |
| Male | 387 (76.9%) | 161 (71.6%) | 226 (81.3%) |  |
| Female | 116 (23.1%) | 64 (28.4%) | 52 (18.7%) |  |
| Marital status |  |  |  | 3.08 |
| Single | 155 (32.7%) | 63 (28.6%) | 92 (36.2%) |  |
| Married | 177 (37.3%) | 87 (39.6%) | 90 (35.4%) |  |
| Separated/divorced/bereaved | 142 (30.0%) | 70 (31.8%) | 72 (28.4%) |  |
| Missing | 29 |  |  |  |
| Employment status |  |  |  | 2.26 |
| Employed | 107 (18.2%) | 41 (18.2%) | 66 (23.7%) |  |
| Unemployed | 396 (81.8%) | 184 (81.8%) | 212 (76.3%) |  |
| Education |  |  |  | 0.44 |
| Less than middle school | 32 (6.7%) | 16 (7.3%) | 16 (6.3%) |  |
| Middle school | 49 (10.3%) | 21 (9.5%) | 28 (11.0%) |  |
| High school | 240 (50.5%) | 105 (47.7%) | 135 (52.9%) |  |
| More than high school | 154 (32.4%) | 78 (35.5%) | 76 (29.8%) |  |
| Missing | 28 |  |  |  |
| Previous hospitalization  due to AUD |  |  |  | 1.01 |
| Yes | 191 (35.6%) | 80 (35.6%) | 111 (39.9%) |  |
| No | 312 (64.4%) | 145 (64.4%) | 167 (60.1%) |  |
| SES† |  |  |  | 0.05 |
| Lower class | 206 (43.3%) | 97 (43.7%) | 109 (42.9%) |  |
| Middle class | 204 (42.9%) | 95 (42.8%) | 109 (42.9%) |  |
| Upper class | 66 (13.9%) | 30 (13.5%) | 36 (14.2%) |  |
| Missing | 27 | 3 |  |  |
| Using the Addiction Support Center^+^ |  |  |  | 2.08 |
| Yes | 104 (20.7%) | 40 (17.8%) | 64 (23.0%) |  |
| No | 399 (79.3%) | 185 (82.2%) | 214 (77.0%) |  |
| Baseline recommended LOC† |  |  |  | 0.98 |
| Level 0.5 | 18 (3.6%) | 7 (3.1%) | 11 (4.0%) |  |
| Level 1 | 99 (19.7%) | 49 (21.8%) | 50 (18.0%) |  |
| Level 2 | 84 (16.7%) | 33 (14.7%) | 51 (18.4%) |  |
| Level 3 | 137 (27.2%) | 74 (32.9%) | 63 (22.7%) |  |
| Level 4 | 165 (32.8%) | 62 (27.6%) | 103 (37.1%) |  |

^+:^: The Addiction Management Integrated Support Center is a community center that supports addicts. Patients visit the center for assistance with diagnosis, economic resources, Alcoholics Anonymous meetings, and rehabilitation.

† The Mantel-Haenszel Chi-square test was used because the row and column variables are on an ordinal scale.
*Note. r* represents the number of categories in the smaller variable of the contingency table (Cohen, 1977).
^*^*p* < .05 ^**^*p* < .01 ^***^*p* < .001
