# Supplementary material for: Associations of the Korean patient placement criteria matching among individuals with alcohol-related problems with treatment completion and abstinence: an observational study
Source: Addict Sci Clin Pract. 2024 Dec 26;19:98. doi: 10.1186/s13722-024-00521-2 (PMC11670465; doi:10.1186/s13722-024-00521-2)
Supplement: Supplementary file 3 — Supplementary Material 3. [file 13722_2024_521_MOESM3_ESM.docx]

Appendix Table 4. Association of KPPC-matched treatment with the number of months of treatment completion with and without adjustment for confounders (Participants whose 1^st^ referral was to outpatient care, *n* =89)

| Model type | Fit statistics | | | Predictor variable | Contrast | Odds ratio  [95% CI] | *p* |
| --- | --- | --- | --- | --- | --- | --- | --- |
|  | AIC | SC | -2LL |  |  |  |  |
| Unadjusted^a^ | 192.72 | 205.16 | 182.72 | No. of PPC-matched treatments | 1 vs. 0 | 8.02 [1.18-54.43] | .03 |
|  |  |  |  |  | 2 vs. 0 | 6.51 [1.27-33.28] | .02 |
| Adjusted^b^ | 193.73 | 213.64 | 177.73 |  | 1 vs. 0 | 5.97 [0.85-41.86] | .07 |
|  |  |  |  |  | 2 vs. 0 | 4.03 [0.73-22.27] | .11 |
|  |  |  |  | Sex | Male vs. female | 0.57 [0.21-1.60] | .29 |
|  |  |  |  | Employment Status | Employed vs. Unemployed | 0.61 [0.18-2.10] | .43 |
|  |  |  |  | Previous hospitalization due to AUD | Yes vs. No | 1.98 [0.82-4.78] | .13 |
| ^a^The unadjusted model includes only one predictor variable, *No. of PPC-matched treatments*.  ^b^The adjusted model adds *Sex,* *Employment status, and Previous hospitalization due to AUD*.  *Note.* Two ordinal logistic regression models did not satisfy the proportional odds assumption at the significance level of 0.01 or lower. The likelihood ratio test based on -2LLs from the two models was not significant, and the model fit statistics, AIC and SC were smaller in the unadjusted model. The wide confidence intervals for the odds ratios of the PPC-matched treatments indicate large standard errors, potentially due to a small sample size. Therefore, the interpretation of the results should be approached with caution. | | | | | | | |

Appendix Table 5. Association of KPPC-matched treatment with the number of months of treatment completion with and without adjustment for confounders (Participants whose 1^st^ referral was to inpatient care, *n*=136)

| Model type | Fit statistics | | | Predictor variable | Contrast | Odds ratio  [95% CI] | *p* |
| --- | --- | --- | --- | --- | --- | --- | --- |
|  | AIC | SC | -2LL |  |  |  |  |
| Unadjusted^a^ | 345.20 | 359.76 | 335.20 | No. of PPC-matched treatments | 1 vs. 0 | 2.85 [1.20-6.81] | .02 |
|  |  |  |  |  | 2 vs. 0 | 4.18 [1.82-9.58] | < .001 |
| Adjusted^b^ | 349.76 | 373.06 | 333.76 |  | 1 vs. 0 | 2.73 [1.14-6.58] | .02 |
|  |  |  |  |  | 2 vs. 0 | 3.77 [1.60-8.92] | < .01 |
|  |  |  |  | Sex | Male vs. female | 0.85 [0.43-1.69] | .65 |
|  |  |  |  | Employment Status | Employed vs. Unemployed | 0.68 [0.29-1.59] | .37 |
|  |  |  |  | Previous hospitalization due to AUD | Yes vs. No | 1.18 [0.60-2.31] | .63 |
| ^a^ The unadjusted model includes only one predictor variable, *No. of PPC-matched treatments*.  ^b^ The adjusted model adds *Sex,* *Employment status, and Previous hospitalization due to AUD*.  *Note.* The adjusted ordinal logistic regression model did not satisfy the proportional odds assumption at the significance level of 0.05, potentially due to a small sample size. The likelihood ratio test based on -2LLs from the two models was not significant, and the model fit statistics, AIC and SC were smaller in the unadjusted model. | | | | | | | |
